# Hepatoprotective Effect of the Penthorum Chinense Pursh Extract against the CCl_4_-Induced Acute Liver Injury via NF-κB and p38-MAPK PATHWAYS in Dogs

**DOI:** 10.3390/ani12050569

**Published:** 2022-02-24

**Authors:** Weilai Tao, Xin Yue, Ruiling Ye, Fazul Nabi, Yangfei Shang, Zhaorong Zhu, Bhutto Zohaib Ahmed, Juan Liu

**Affiliations:** 1 College of Veterinary Medicine, Southwest University, Chongqing 402460, China; ttaoweilai@163.com (W.T.); walterlaansel@gmail.com (X.Y.); 13350557869@163.com (R.Y.); fazulnabishar@yahoo.com (F.N.); 13255968963@163.com (Y.S.); 15708928972@163.com (Z.Z.); 2 Chinese Veterinary Herbal Drugs Innovation Research Lab, University Veterinary Science Engineering Research Center in Chongqing, Chongqing 402460, China; 3 Immunology Research Center of Medical Research Institute, Southwest University, Chongqing 402460, China; 4 Faculty of Veterinary and Animal Sciences, Lasbela University of Agriculture, Water, and Marine Sciences, Uthal 90150, Pakistan; bhutto92zohaib@gmail.com

**Keywords:** acute liver injury, Penthorum Chinense Pursh, MAPK/NF-κB signaling, oxidative stress, inflammation

## Abstract

**Simple Summary:**

Traditional Chinese herbal medicines have been used to treat animal diseases and play an important role in the treatment of liver injury. The current study used Penthorum Chinense Pursh extract (PCPE) to treat acute liver injury of dogs caused by CCl_4_. Our study found that PCPE reduced the pathological symptoms and liver tissue lesions caused by ALI, improved serum biochemical indicators, and alleviated the inflammatory response and oxidative stress. Our results clarified that the NF-κB and MAPK signaling pathways in dogs are related to the antioxidant and anti-inflammatory effects of PCPE. PCPE may be used as a safe and effective medicine for the treatment of acute liver injury in dogs.

**Abstract:**

Acute liver injury (ALI), manifested by acute hepatocellular damages and necrosis, is a life-threatening clinical syndrome and Penthorum Chinense Pursh (PCP) is a well-known folk medicine practiced for liver-related diseases. This study aimed to investigate the ameliorative effects of PCP extract (PCPE) on carbon tetrachloride (CCl_4_) induced ALI in dogs via mitogen-activated protein kinase (MAPK) and Nuclear factor κB (NF-κB) signaling pathway. Healthy dogs were induced by CCl_4_ and treated with different dosage regimes of PCPE for 7 days. CCl_4_ produced acute liver injury and induced both oxidative stress and an inflammatory response in dogs. The PCPE significantly ameliorated and improved vacuolar inflammatory lesions in liver tissues during ALI, enhanced activity of superoxide dismutase, and restored glutathione peroxidase, further significantly reducing the indices of malondialdehyde and nitric oxide in serum. Inflammatory factors (IL-1β, IL-6, and TNF-α) were declined and anti-inflammatory factors (IL-10) were increased by the application of PCPE. PCPE treatment, down-regulated the MEKK4, MKK3, p38MAPK, MSK1, and NF-κB, and upregulated the IkB mRNA levels (*p* < 0.01) in ALI affected dogs. In conclusion, PCPE repaired acute liver injury by improving antioxidant enzymes and by reducing oxidation products. Furthermore, the PCPE inhibited the MAPK/NF-κB signaling pathway, which resulted in anti-inflammatory and antioxidant effects on ALI-induced dogs. In the future, PCPE could be a useful ethnomedicine in veterinary clinical practices for the treatment of liver injuries or failures.

## 1. Introduction

The liver is a vital organ involved in the metabolism and detoxification of xenobiotics, chemicals, drugs, and foreign particles from the body. Acute liver injury (ALI) is a common disease of the canine family that is caused by diverse factors including drug poisoning, alcohol abuse, radioactive damage, vascular disorders, and other exogenous substances, finally leading to abnormal liver functions [1]. ALI pathogenesis is controlled by a variety of mechanisms and increases both the inflammatory response and oxidative stress, resulting in morbidity and mortality without being cured [2,3]. Carbon tetrachloride (CCl_4_) was reported for its hepatotoxic effects, and is a widely used experimental chemical for the induction of ALI resulting in liver injury and oxidative stress [4].

Acute liver injuries or failure caused by CCl_4_-induction are associated with oxidative stress and inflammatory responses [5]. It is well established after reported studies that mitogen-activated protein kinase (MAPK) and nuclear factor kappa B (NF-κB) signaling pathways play an important role in controlling the ALI via protective mechanisms which include inflammatory response and oxidative stress [6]. The oxidative stress involved in the pathogenesis of liver injury as an intracellular serine or threonine protein kinase, MAPK can be activated by stress factors and inflammatory stimulation and plays an important role in cell proliferation, differentiation, and apoptosis [7,8]. Moreover, oxidative stress has also been reported to activate NF-κB, an important transcriptional regulator in cells, and induces NF-κB nuclear translocation to promote the expression of pro-inflammatory genes, including tumor necrosis factor α (TNF-α), Interleukin-1β (IL-1β), Interleukin-6 (IL-6) [9,10]. Therefore, the p38MAPK and NF-κB signaling pathways have been proposed as targets of the therapeutic pathway of ALI.

Recently, researchers are more focused on herbal or folk medicine for the treatment of acute liver diseases due to curative and low toxic effects [11]. Penthorum Chinense Pursh is an edible plant well-known for its usage in Chinese traditional medicine practice, reported for its therapeutic effects on infectious hepatitis, edema, and various liver diseases [12,13]. It is reported that Penthorum Chinense Pursh Extract (PCPE) has various pharmacological effects, such as anti-inflammation and anti-oxidation, and can improve liver injuries by eliminating oxygen free radicals and inhibiting inflammatory response [14,15]. These pharmacological effects are closely related to the active components of PCPE. Flavonoids, organic acids, coumarins, lignans, polyphenols, and sterols are important bioactive constituents of PCPE, and an abundance of research has demonstrated that many extracts have significant pharmacological activities of anti-inflammation and anti-oxidation [13,16].

In veterinary clinical practice, few studies have been conducted regarding the effects of PCPE and its underlying signaling mechanisms for acute liver injury, while most studies were conducted in human and cell lines [17,18]. This highlighted the necessity of further investigation of PCPE effects and related signaling mechanisms involved with acute liver injury in dogs. 

Recently, studies have shown that aqueous extract of PCP and their compositions could protect against the acute and chronic CCl_4_ and alcohol-induced liver injuries in mice through ameliorating oxidative stress and activating Nrf2 signaling pathways respectively [18,19]. There are very limited studies on the effect of PCPE on CCI_4_ liver damages in dogs by inhibition of MAPK and NF-κB-mediated inflammatory and oxidative signaling pathways. In the present study, the CCl_4_- ALI model was established for exploring the mechanisms of PCPE medicine in reversing ALI in dogs. The protective effect might be associated with the reduced inflammatory response and oxidative stress via MAPK/NF-κB signaling pathways, however, further underlying mechanisms with respect to Autoimmune hepatitis disease are suggested for future studies.

## 2. Materials and Methods

### 2.1. Experiment Design and Sample Collection

48 Chinese Rural Dogs with 2–4 months of age and weighing 2–3 Kg were provided by the Experimental Animals Department of Southwest University (Rongchang Chongqing). Experimental animals were managed and handled (30 °C, 56–60% humidity, *ad libitum* food and water) according to principles approved by the Institutional Animal Care and Use Committee, Southwest University Chongqing, China (Approval no. IACUC-20180413). Dogs were categorized into six groups (n = 8, male and female). Group-I without treatment served as control, Group-II treated with CCl_4,_ Group-III treated with Biphenyl Dimethyl Dicarboxylate Pills (BDDP), Group-IV, V, and VI were treated with different dosages (high-H, medium-M, low-L) of PCPE. 

Firstly, dogs were divided into control and CCl_4_-induced groups. Dogs were injected 50% CCl_4_ peanut oil-solution (1 ml/Kg body weight) subcutaneously in the neck for two days to establish a canine liver injury. After the establishment of CCl_4_-induced ALI, Biphenyl Dimethyl Dicarboxylate Pills (positive drug-BDDP group), and PCPE high, medium, and low dosages (1.5, 1, 0.5 herb mL^−1^, respectively) were administered through gastric lavage twice a daily for 7 days till the end of the experiment. The herbal dosages of PCPE were measured based on human and animal body weight and according to Research and Evaluation of Pharmacodynamics [20]. During the experimental period daily basis clinical symptoms were recorded and monitored. The serum was withdrawn from blood samples collected from the brachiocephalic vein during the 3rd, 5th, and 7th days. And each dog was euthanized by an intravenous overdose of pentobarbital, and then liver samples were collected on the 7th day. 

### 2.2. Preparation of PCPE

Penthorum Chinense Pursh grass was purchased from Zhongmiao Pharmaceutical Co., Ltd. (provided by Gulin, Luzhou, Sichuan Province, China). The phenotypical characteristic of grass was identified by Botanist Dr. Liu Juan from Southwest University. Grass pieces were decocted with 4000 mL of 70% ethanol solution and soaked for 4 hours, and the reflux extraction process was carried out for 90 min before filtrate was obtained. This step was repeated two times, filtrate was added 3 times after the combination of the concentrate the filter was decompressed in the rotary vacuum evaporator at 80 °C, and subsequently concentrated to 1.5 g of herb mL^−1^, and stored at 4 °C.

### 2.3. Identification of Active Ingredients of PCPE by Infrared Spectroscopy

PCPE powder was mixed with a Potassium bromide wafer (KBr wafer), comminuted, and then pressed into a tablet for measurement, and infrared spectrum scanning was performed in the range of 4000 cm^−1^ to 400 cm^−1^.

### 2.4. Criteria for Judging Efficacy 

The clinical signs (mental state, exercise posture, behavior, drinking, feeding, feces/urine) of CCl_4_-induced dogs (ALI dogs) were monitored daily as the curative standard of PCPE therapy to ALI in dogs. ALI dogs or CCl_4_-induced dogs showed clinical signs including depression, weakness, loss of appetite, decreased mobility, significant wasting, choroidal congestion in the eye, yellowing of mucous membranes, abdominal bloating, oliguria with yellow urine. The clinical examination was performed after the administration of PCPE resulted in the disappearance and improvement of symptoms [21]. Finally, effective rate, cure rate, and improvement rate were calculated according to the following formula:

(1) $$\mathrm{cure}\;\mathrm{rate}\;\left(\%\right)=\frac{Number\;of\;animals\;with\;disappeared\;symptoms}{Number\;of\;sick\;animals}\;\%$$

(2) $$\mathrm{improvement}\;\mathrm{rate}\;\left(\%\right)=\frac{Number\;of\;animals\;with\;improved\;symptoms}{Number\;of\;sick\;animals}\;\%$$

(3)  effective rate = cure rate+ improvement rate

### 2.5. Histopathology of Liver

After the fixation, the liver tissues were washed, dehydrated, waxed, embedded, sectioned, and counterstained in a dehydration box using a standard method. The well-stained liver tissue sections were selected and the histopathological changes of the liver-stained sections were observed under an optical microscope at 400× magnification (Zeiss upright microscope Axio Scope A1; Carl Zeiss, Oberkochen, Germany).

### 2.6. Biochemical Assay

The content of total bile acid (TBA) and Alanine aminotransferase (ALT), Aspartate aminotransferase (AST), and γ-glutamyl transpeptidase (GGT) activity in canine serum were detected by automatic biochemical analysis according to the manufactures kit instructions (Beckman Coulter AU680, Beckman, America).

### 2.7. Oxidative and Inflammatory Assay

The oxidative stress markers, including superoxide dismutase (SOD), Plasma glutathione peroxidase (GSH-Px), malondialdehyde (MDA) and Nitric Oxide (NO), and inflammation-related factors, including in serum were measured by enzyme-linked immunosorbent assay (ELISA) according to the manufacture’s kit instructions (Xiamen Jiahui Biotechnology Co., Ltd., Xiamen, China).

### 2.8. RT-qPCR

About 50 mg of liver tissue was taken from the control, CCL_4_, BDDP, and PCPE-H groups. Total RNA was extracted according to the manufacturer’s instructions using an RNA extraction kit (Sangon Biotech Shanghai Co., Ltd., Shanghai, China). Total RNA was used as the amplification template for reverse transcription amplification of cDNA using a Trans Gen cDNA kit (Biotech Co., Ltd., Beijing, China), and stored at −20 °C. The mRNA primers of different genes were synthesized by Dalian Bao Biological engineering Co., Ltd. (Table 1). The real-time PCR reaction was conducted according to the kit instructions (TransGen Biotech, Beijing, China), and relative mRNA transcription volume was calculated by 2^−^^△△Ct^ [22].

### 2.9. Statistical Analysis

Data were analyzed by one-way ANOVA using SPSS 20.0.0 (IBM, Armonk, NY, USA). All parameters determined in this study are presented as the mean ± standard deviation (ns: *p* > 0.05; *: *p* < 0.05; **: *p* < 0.01). GraphPad Prism version 8.0.1 (GraphPad Software, San Diego, CA, USA) was used to generate graphs with error bars.

## 3. Results

### 3.1. Infrared Spectroscopy Analysis

**Figure 1:**
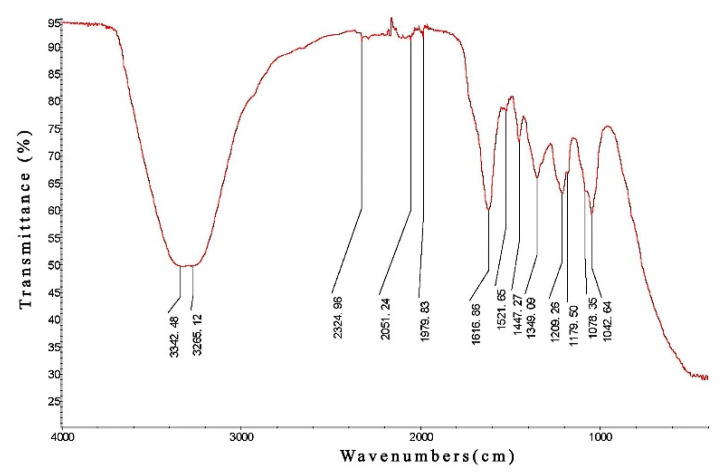
Infrared Spectroscopy (IR spectrum) of PCPE.

The infrared spectrum results showed that the absorption peak was relatively dense in the band of 2000–900 cm^−1^, and the band of 1310–900 cm^−1^ was the stretching vibration region of a single bond and the bending vibration of some hydrogen-containing groups. 1500–1310 cm^−1^ mainly represents the bending vibration of C-H. The band of 2000–1500 cm^−1^ is mainly characterized by carbon skeleton ring respiratory vibration of the benzene ring and stretching vibration of C=N, C=O, and C=C. The bands of PCPE in the region 2000–900 cm^−1^ are similar to those of kaempferol, quercetin, and myricetin which the IR spectra are dominated by the bands in the region 1800–1000 cm^−1^ [23]. 3400–3200 cm^−1^ is mainly O-H stretching vibration, which indicates that PCPE mainly contains flavonoid active components (Figure 1). Many flavonoids such as quercetin and kaempferol have been demonstrated to be the important bioactive constituents of Penthorum Chinense Pursh and possess strong hepatoprotective activity [13].

### 3.2. Effect of PCPE on Clinical Symptoms of ALI Dogs

The results showed that the improvement rate and effective rate of the PCPE-H group were 62.5% and 87.5%, and were 50% and 62.5% of the PCPE-M group, whereas the PCPE administration in the PCPE-L group does not show improvement in clinical signs (Table 2).

### 3.3. Histopathological of Liver Tissues

**Figure 2:**
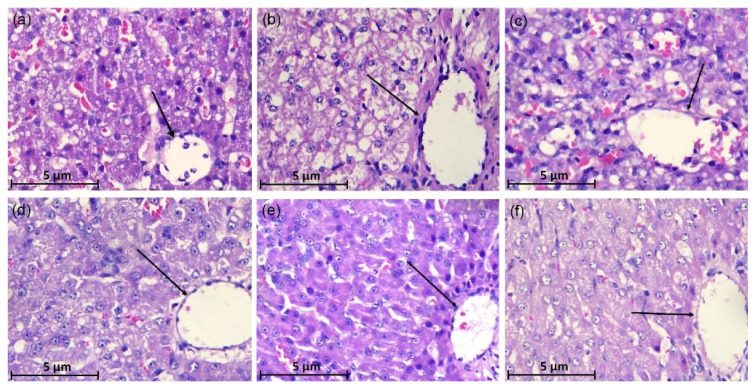
Histopathology of Acute Liver Injury in dogs. control (**a**), CCl_4_ (**b**), BDDP (**c**), PCPE-H (**d**), PCPE-M (**e**) and PCPE-L (**f**) treatments (HE, 400×). The arrows indicate the canine liver portal area in the figure.

In the CCl_4_-induced group, canine hepatocytes showed degeneration, swelling, and necrosis, with a disordered arrangement of hepatic cords, a large number of inflammatory cells infiltrated, and the cytoplasm was almost transparent, forming vacuoles (Figure 2b). As compared with the CCl_4_ group the degeneration of canine hepatocytes in the BDDP group was improved, the degree of injury was reduced, and the liver tissues were orderly arranged, but vacuolar degeneration was still observed (Figure 2c). As compared to the CCl_4_-induced group, PCPE reversed the ALI in a dose-dependent (high to low, respectively) manner by improved hepatocytes degeneration, reduction in cell swelling, and reduction in inflammatory cell infiltration (Figure 2d–f).

### 3.4. Biochemical Analysis of Serum

**Figure 3:**
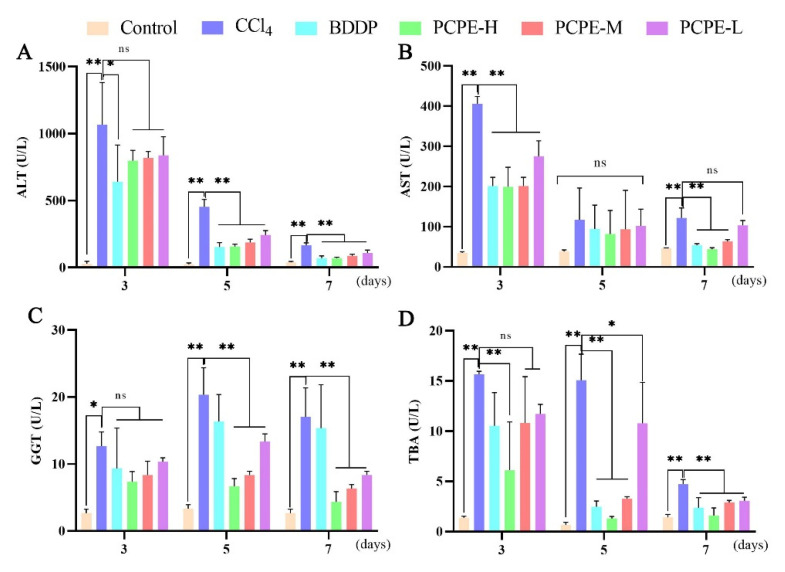
PCPE effects on liver function enzymes in CCl_4_-induced ALI dogs. (**A**) ALT, (**B**) AST, (**C**) GGT, (**D**) TBA. Data represented as Means ± SD. *, **, and ns indicates *p* < 0.05, *p* < 0.01, non-significant, respectively.

The effects of CCl_4_ and PCPE on hepatic functional enzymes (ALT, AST, GGT, and TBA) in serum were quantified on the 3rd, 5th, and 7th day of treatment. The significantly increased indices of ALT, AST, GGT, and TBA were observed in the serum of the CCl_4_ vs. control. In comparison with CCl_4_ induced group, PCPE ameliorated and improved the adverse effects by decreasing the levels of ALT, AST, GGT, and TBA in serum (Figure 3).

### 3.5. Serum Antioxidant Indexes

**Figure 4:**
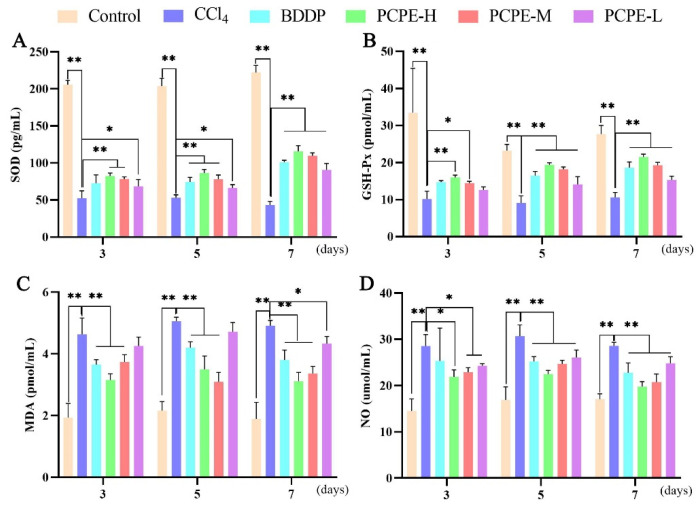
PCPE effects on serum antioxidant indices. (**A**) SOD, (**B**) GSH-Px, (**C**) MDA, (**D**) NO. Data represented as Means ± SD. * and ** indicates *p* < 0.05, *p* < 0.01, respectively.

Indices of SOD and GSH-Px in the serum were decreased, and indices of MDA and NO were increased in the CCl_4_ group vs. control group on the 3rd, 5th, and 7th days (*p* < 0.01). In comparison with the CCl_4_ group, however, the activity of SOD and GSH-Px were increased and the content of MDA and NO decreased in the BDDP and all PCPE treated groups (*p* < 0.01). The PCPE-H produced more impact on antioxidant enzymes indices on the 7th day (Figure 4).

### 3.6. Analysis of Inflammatory Cytokine

**Figure 5:**
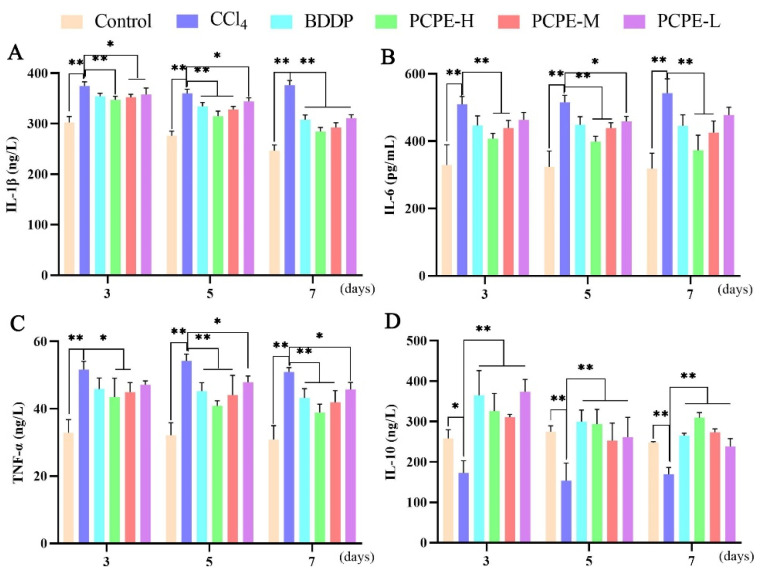
PCPE effects on inflammatory cytokines in dogs with ALI induced by CCl_4_. (**A**) IL-1β, (**B**) IL-6, (**C**) TNF-α, (**D**) IL-10. Data represented as Means ± SD. * and ** indicates *p* < 0.05, *p* < 0.01, respectively.

Essential serum pro-inflammatory cytokines (IL-1β, IL-6, and TNF-α) and anti-inflammatory cytokines (IL-10) expressions in CCl_4_-induced liver injury were measured. The serum levels of IL-1β, IL-6, and TNF-α in the CCl_4_ group were significantly increased (*p* < 0.01), and IL-10 were decreased on the 3rd, 5th, and 7th day of treatment compared to those in the control group. After administration of BDDP and PCPE decreased in the levels of pro-inflammatory cytokine and an increase in the level of anti-inflammatory cytokine was observed in CCl_4_-induced liver injury (Figure 5).

### 3.7. PCPE Effects on Transcription of MAPK/NF-κB

**Figure 6:**
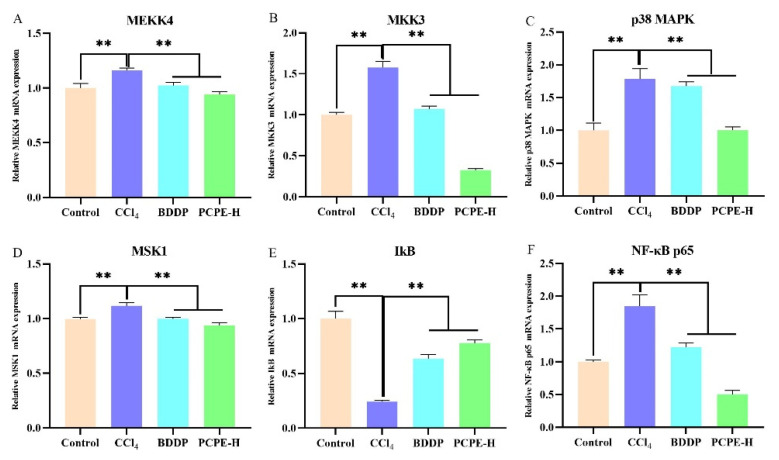
PCPE effects on MAPK/NF-κB signaling pathways. (**A**) MEKK4, (**B**) MKK3, (**C**) p38MAPK, (**D**) MSK1, (**E**) IkB, (**F**) NF-κB p65. Data represented as Means ± SD. ** indicates *p* < 0.01.

The serum indexes revealed that PCPE-H had a greater influence on improving ALI. For further validation, PCPE effects were explored on signaling pathways through qRT-PCR. In contrast with control, MEKK4, MKK3, p38MAPK, MSK1, and NF-κB p65 genes were overexpressed, whilst IkB was decreased in the CCl_4_-induced group (*p* < 0.01). However, reciprocal effects were observed in mRNA levels (*p* < 0.01) by administrating BDDP and PCPE-H against CCl_4_-induced ALI (Figure 6).

## 4. Discussion

The pathogenesis of acute or chronic liver injury is governed by oxidative stress in various animal species [24]. The over-generation of reactive oxygen species (ROS) may be induced by various hepato-toxicants including metals, alcohol, or CCl_4_. CCl_4_ is commonly used for establishing an experimental model of acute liver injury (ALI) [4], so novel ethnomedicine or allopathic medicine will be investigated. CCl_4_ is metabolized by the liver and triggers free radical production, such as the trichloromethyl group [25]. The generated free radicals can directly induce lipid peroxidation in the cell membrane and destroy the cell membrane, which is damaged by free radical chain reaction, and the hepatocytes produce oxidative stress, degeneration, hepatocellular injury, and necrosis finally by the induction of ALI [26].

Penthorum Chinense Pursh is a traditional Chinese rich with active constituents such as flavonoids, organic acids, and terpenoids, and are commonly used for liver diseases with remarkable curative and low toxic effects [27]. Some studies revealed that 5-hydroxy-flavanone-7-O-β-D-glucoside, quercetin, kaempferol, pinocembrin, catechins, and so on are important constituents of Penthorum Chinense Pursh extracts [28,29]. Most constituents of them are flavonoids. We also validated through infrared spectroscopy that flavonoids and polyphenols were rich therapeutic compounds in PCPE and might be involved in the hepatoprotective effect. For instance, kaempferol, quercetin, Pinocembrin-7-O-beta-D-glucoside, and many other flavonoids and phenolic compounds, extracted from Penthorum Chinense Pursh, have been reported that they have strong antioxidant and antiinflammation activities, and have significant liver-protecting effects [30,31].

The present study evaluates the protective effect of PCPE against CCl_4_-induced ALI in dogs and which is linked with the NF-kB and MAPK pathways. Infrared microscopy results demonstrated that flavonoids (biomolecule compounds) in more concentration were found in the PCPE. It is speculated that reversing the effects on ALI such as interaction with liver histology, liver function enzymes, antioxidant enzymes, inflammatory cytokines, and MAPK/NF-κB pathways might be due to flavonoids. Previous reports suggest that flavonoids interacted with inflammatory mediators, and MAPK/NF-κB pathways [32,33,34].

The liver is the main organ for numerous physiological processes, such as macronutrient metabolism, endocrine, and exocrine functions, immune response, growth and development, and the breakdown of xenobiotic compounds [35]. The integrity of the liver tissue structure is a prerequisite to maintaining normal function [36]. Liver functional indicators are a variety of enzymes such as amino transaminases [37]. As a result of liver cells damage due to toxicity, the functional activity has lost the lead to an increase in releasing of biomarkers or enzymes (AST and ALT) into the blood [38]. During the present study, the liver structure is damaged and the level of liver function enzymes was abnormal owing to CCl_4_-mediated injuries, indicating that liver functions were impaired, however, liver function significantly improved with PCPE treatment.

Oxidative stress is considered to be a key factor leading to liver damage. SOD and GSH-Px are important antioxidant enzymes in the body, which can remove important free radicals in the body, inhibit lipid peroxidation and protect liver cells [39]. MDA is the product of lipid peroxidation induced by oxidative stress [40]. NO is the product of the internal nitrogen-free radical and nitrogen-containing small molecule metabolism process, which can reflect the degree of oxidative stress [41]. Our results show that PCPE improved CCl_4_-induced oxidative stress by producing an impact on antioxidant and oxidative stress-related enzymes (SOD and GSH-Px, MDA, and NO). 

Inflammatory reactions occur around the liver tissue in ALI dogs and play a key role in controlling liver disease as previously reported [42]. Pro-inflammatory factors such as IL-1β, IL-6, and TNF-α are important mediators involved in the inflammatory response, and IL-10, a multifunctional negative anti-inflammatory factor, acts as an antagonistic inflammatory mediator [43,44]. IL-1β is a member of the IL-1 family and is a central cytokine in many diseases. IL-1β exerts a pro-inflammatory role in liver disease by interacting with IL-1R and the expression is low in the healthy liver [45]. IL-6 is mainly produced by pro-inflammatory cells and hepatocytes and has both anti-inflammatory and anti-inflammatory biological effects. IL-6 can promote hepatocyte survival and regeneration in some ALI models [46]. But many studies suggested that IL-6 promoted liver inflammation and aggravated liver damage in CCl4-induced injury [47,48]. TNF-α is primarily produced by activated macrophages and plays a crucial role in activating NF-κB and MAPK pathways, and then results in systemic inflammation. ALI is closely related to the increasing level of TNF-α [49,50]. IL-10 can be produced by lymphocytes and inhibit liver fibrosis [51]. IL-10 has also been reported to play an anti-inflammatory role in acute liver injury. Phytochemical extracted from herbs regulate the release of these inflammation-related cytokines and play an important role in the treatment of disease [52]. We validated that pro-inflammatory factors (IL-1β, IL-6, and TNF-α) were reduced and anti-inflammatory factors were increased after administration of PCPE in challenged (CCl_4_-induced) leading to reversing in ALI. 

**Figure 7:**
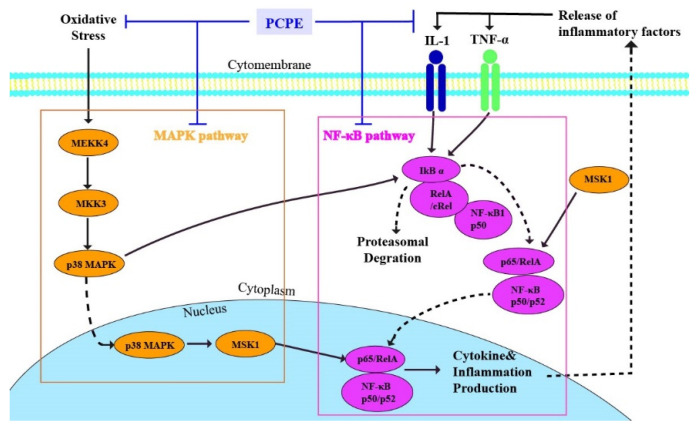
PCPE interactions with MAPK/NF-κB signaling pathway during ALI in dogs.

MAPK signaling transmits upstream signals to downstream response molecules by sequential phosphorylation [53]. MEKK4 is a member of the MEKKK protein kinase family. Phosphorylated MEKK4 activates MKK3, which regulates the expression of MEKK4, and phosphorylated MKK3 activates p38MAPK [54]. Phosphorylated p38MAPK regulates the transcription of downstream genes and plays its biological function, such as regulating the inflammatory response [55]. The activated p38MAPK was transcribed into the nucleus and activated its downstream nuclear protein kinase MSK1, which eventually activated NF-κB [56]. The NF-κB signaling pathway is a key pathway involved in immune and inflammatory responses [57], and IkB is a negative regulatory protein of NF-κB [58]. In the present study, we mainly explored the effect of PCPE on ALI inducing by CCl_4_ via MAPK/NF-κB pathway. Our findings indicate that CCl_4_ activated MAPK/NF-κB pathway. However, PCPE treatment inhibited the overexpression of MAPK/NF-κB pathway-related genes (MEKK4, MKK3, p38 MAPK, MSK1, and NF-κB) induced by CCl_4_, whereas upregulation of IkB was observed. Therefore, PCPE treatment can reduce the inflammatory response and oxidative damage by suppressing MAPK and NF-κB signaling pathways, thereby improving ALI. (Figure 7)

## 5. Conclusions

In conclusion, PCPE treatment effectively improved CCl_4_-induced ALI by increasing anti-inflammatory and by reducing pro-inflammatory factors. This protective effect of PCPE is linked with the reduced oxidative stress and the enhanced oxidant defense systems via the suppression of MAPK/NF-κB Signaling Pathway. Our results suggest that Penthorum Chinese Pursh Extract can be used in veterinary clinical practices as an effective herbal drug for the treatment of acute liver injury.

## Figures and Tables

**Table 1:** Primers for quantitative real-time PCR.

Target Gene	Primer Sequence (5′-3′)	Product Length (bp)
MEK4	F: ATGGGAGCTTTGCCTTTGTTA R: GCTCCATTATCCGCTTTACCTG	21 22
MKK3	F: AAGCCACCTGTACCCAACC R: GGCATGTCGCACCTTCTCTAC	19 21
P38 MAPK	F: TTGGACTGGCCCGACATAC R: GCCATTATGCATCCCACTGAC	19 21
MSK1	F: GGTTAGTGGCCTAGCACGAC R: AAACCTGCGACCGACTCAGTA	20 21
NF-κB p65	F: GCGCTTGTCACCTGTCCTC R: AGCCTGGTCCCGTGAAATAC	19 21
IκB	F: GGCCATCACGAGAGATCAATG R: TCCTGTTGGTAGCGGTAGAAG	21 21
GAPDH	F: ATGGTGAAGGTCGGAGTGAA R: GGAATTTGCCGTGGGTAGAAT	20 21

**Table 2:** Effect of PCPE on ALI dogs.

Group	Numbers	Cure Rate (%)	Improvement Rate (%)	Efficient Rate (%)
Control	8	-	-	-
CCl_4_	8	-	-	-
BDDP	8	12.5	62.5	75
PCPE-H	8	25	62.5	87.5
PCPE-M	8	12.5	50	62.5
PCPE-L	8	0	37.5	37.5

## Data Availability

Not applicable.

## References

[1] Kim S.R., Park E.J., Dusabimana T., Je J., Jeong K., Yun S.P., Kim H.J., Cho K.M., Kim H., Park S.W. (2020). *Platycodon grandiflorus* Fermented Extracts Attenuate Endotoxin-Induced Acute Liver Injury in Mice. Nutrients.

[2] Ning C., Gao X., Wang C., Huo X., Liu Z., Sun H., Yang X., Sun P., Ma X., Meng Q. (2018). Hepatoprotective effect of ginsenoside Rg1 from Panax ginseng on carbon tetrachloride-induced acute liver injury by activating Nrf2 signaling pathway in mice. Environ. Toxicol..

[3] Zhang X., Kuang G., Wan J., Jiang R., Ma L., Gong X., Liu X. (2020). Salidroside protects mice against CCl4-induced acute liver injury via down-regulating CYP2E1 expression and inhibiting NLRP3 inflammasome activation. Int. Immunopharmacol..

[4] Zhao L., Jin Y., Donahue K., Tsui M., Fish M., Logan C.Y., Wang B., Nusse R. (2019). Tissue Repair in the Mouse Liver Following Acute Carbon Tetrachloride Depends on Injury-Induced Wnt/β-Catenin Signaling. Hepatology.

[5] Munakarmi S., Chand L., Shin H.B., Jang K.Y., Jeong Y.J. (2020). Indole-3-Carbinol Derivative DIM Mitigates Carbon Tetrachloride-Induced Acute Liver Injury in Mice by Inhibiting Inflammatory Response, Apoptosis and Regulating Oxidative Stress. Int. J. Mol. Sci..

[6] Ko I.G., Jin J.J., Hwang L., Kim S.H., Kim C.J., Han J.H., Lee S., Kim H.I., Shin H.P., Jeon J.W. (2020). Polydeoxyribonucleotide Exerts Protective Effect Against CCl_4_-Induced Acute Liver Injury Through Inactivation of NF-κB/MAPK Signaling Pathway in Mice. Int. J. Mol. Sci..

[7] Qian S., Li C., Liu X., Jia X., Xiao Y., Li Z. (2021). Activation of the JNK/MAPK Signaling Pathway by TGF-β1 Enhances Neonatal Fc Receptor Expression and IgG Transcytosis. Microorganism.

[8] Yin P., Zhang Z., Li J., Shi Y., Jin N., Zou W., Gao Q., Wang W., Liu F. (2019). Ferulic acid inhibits bovine endometrial epithelial cells against LPS-induced inflammation via suppressing NK-κB and MAPK pathway. Res. Vet. Sci..

[9] Xie C., Li X., Zhu J., Wu J., Geng S., Zhong C. (2019). Magnesium isoglycyrrhizinate suppresses LPS-induced inflammation and oxidative stress through inhibiting NF-κB and MAPK pathways in RAW264.7 cells. Bioorg. Med. Chem..

[10] Canty T.G., Boyle E.M., Farr A., Morgan E.N., Verrier E.D., Pohlman T.H. (1999). Oxidative stress induces NF-κB nuclear translocation without degradation of IκBα. Circulation.

[11] Ali M., Khan T., Fatima K., Ali Q.U.A., Ovais M., Khalil A.T., Ullah I., Raza A., Shinwari Z.K., Idrees M. (2018). Selected hepatoprotective herbal medicines: Evidence from ethnomedicinal applications, animal models, and possible mechanism of actions. Phytother. Res. PTR.

[12] Wang A., Lin L., Wang Y. (2015). Traditional Chinese Herbal Medicine Penthorum chinense Pursh: A Phytochemical and Pharmacological Review. Am. J. Chin. Med..

[13] Wang A., Li M., Huang H., Xiao Z., Shen J., Zhao Y., Yin J., Kaboli P.J., Cao J., Cho C.H. (2020). A review of Penthorum chinense Pursh for hepatoprotection: Traditional use, phytochemistry, pharmacology, toxicology and clinical trials. J. Ethnopharmacol..

[14] Jeong D., Lee J., Park S.H., Kim Y.A., Park B.J., Oh J., Sung G.-H., Aravinthan A., Kim J.-H., Kang H. (2019). Antiphotoaging and Antimelanogenic Effects of Penthorum chinense Pursh Ethanol Extract due to Antioxidant- and Autophagy-Inducing Properties. Oxid. Med. Cell. Longev..

[15] Zhao W.W., Guo W., Guo J.F., Wang X., Chen X.Q., Wu X. (2021). Three new flavonoids from Penthorum chinense Pursh and their docking studies. Nat. Prod. Res..

[16] Sun Y., He L., Wang W., Wang T., Hua W., Li T., Wang L., Gao T., Chen F., Tang L. (2021). Polyphenols from Penthorum chinense Pursh. Attenuates high glucose-induced vascular inflammation through directly interacting with Keap1 protein. J. Ethnopharmacol..

[17] Zhang T.T., Xu X.L., Jiang M.H., Jiang J.G. (2013). Hepatoprotective function of Penthorum chinense Pursh. Food Funct..

[18] Wang M., Zhang X.J., Feng R., Jiang Y., Zhang D.Y., He C., Li P., Wan J.B. (2017). Hepatoprotective properties of Penthorum chinense Pursh against carbon tetrachloride-induced acute liver injury in mice. Chin. Med..

[19] Cao Y.W., Jiang Y., Zhang D.Y., Wang M., Chen W.S., Su H., Wang Y.T., Wan J.B. (2015). Protective effects of Penthorum chinense Pursh against chronic ethanol-induced liver injury in mice. J. Ethnopharmacol..

[20] Blanchard O.L., Smoliga J.M. (2015). Translating dosages from animal models to human clinical trials—Revisiting body surface area scaling. FASEB J. Off. Publ. Fed. Am. Soc. Exp. Biol..

[21] Wenie Z., Jianqin X. (2014). Zhongshouyixue.

[22] Nabi F., Shahzad M., Liu J., Li K., Han Z., Zhang D., Iqbal M.K., Li J. (2016). Hsp90 inhibitor celastrol reinstates growth plate angiogenesis in thiram-induced tibial dyschondroplasia. Avian Pathol..

[23] Machado N.F.L., de Carvalho L.A.E.B., Otero J.C., Marques M.P.M. (2013). A conformational study of hydroxyflavones by vibrational spectroscopy coupled to DFT calculations. Spectrochim. Acta Part A Mol. Biomol. Spectrosc..

[24] Farzaei M.H., Zobeiri M., Parvizi F., El-Senduny F.F., Marmouzi I., Coy-Barrera E., Naseri R., Nabavi S.M., Rahimi R., Abdollahi M. (2018). Curcumin in Liver Diseases: A Systematic Review of the Cellular Mechanisms of Oxidative Stress and Clinical Perspective. Nutrients.

[25] Ogaly H.A., Eltablawy N.A., El-Behairy A.M., El-Hindi H., Abd-Elsalam R.M. (2015). Hepatocyte Growth Factor Mediates the Antifibrogenic Action of Ocimum bacilicum Essential Oil against CCl4-Induced Liver Fibrosis in Rats. Molecules.

[26] Ma J.-Q., Li Z., Xie W.-R., Liu C.-M., Liu S.-S. (2015). Quercetin protects mouse liver against CCl4-induced inflammation by the TLR2/4 and MAPK/NF-κB pathway. Int. Immunopharmacol..

[27] Du Y.C., Lai L., Zhang H., Zhong F.R., Cheng H.L., Qian B.L., Tan P., Xia X.M., Fu W.G. (2020). Kaempferol from-Penthorum chinense-Pursh suppresses HMGB1/TLR4/NF-κB signaling and NLRP3 inflammasome activation in acetaminophen-induced hepatotoxicity. Food Funct..

[28] Feng H., Wang Z.M., Dong G.Y., Wu Z. (2001). Studies on chemical constitutents from Penthorum chinense Pursh. China J. Chin. Mater. Med..

[29] Ming F., Lin W., Juan Y., Zhaotun H. (2013). Chemical Constituents of Penthorum chinense Pursh. Chin. Pharm. J..

[30] He L., Zhang S., Luo C., Sun Y., Lu Q., Huang L., Chen F., Tang L. (2018). Functional Teas from the Stems of Penthorum chinense Pursh.: Phenolic Constituents, Antioxidant and Hepatoprotective Activity. Plant Foods Hum. Nutr..

[31] Guo W.W., Qiu F., Chen X.Q., Ba Y.Y., Wang X., Wu X. (2016). In-vivo absorption of pinocembrin-7-O-β-D-glucoside in rats and its in-vitro biotransformation. Sci. Rep..

[32] Frattaruolo L., Carullo G., Brindisi M., Mazzotta S., Bellissimo L., Rago V., Curcio R., Dolce V., Aiello F., Cappello A.R. (2019). Antioxidant and Anti-Inflammatory Activities of Flavanones from Glycyrrhiza glabra L. (licorice) Leaf Phytocomplexes: Identification of Licoflavanone as a Modulator of NF-kB/MAPK Pathway. Antioxidants.

[33] Kim H.P., Son K.H., Chang H.W., Kang S.S. (2004). Anti-Inflammatory Plant Flavonoids and Cellular Action Mechanisms. J. Pharmacol. Sci..

[34] Nori S.L., Aquino R.P., Nicolin V., Santoro A. (2015). Flavonoids and flavonoid-rich natural extracts inhibit cytokine release in cystic fibrosis bronchial epithelial cells by regulating NF-kB pathway. Ital. J. Anat. Embryol..

[35] Trefts E., Gannon M., Wasserman D.H. (2017). The liver. Curr. Biol..

[36] Schwabe R.F., Luedde T. (2018). Apoptosis and necroptosis in the liver: A matter of life and death. Nat. Rev. Gastroenterol. Hepatol..

[37] Ding H.-R., Wang J.-L., Ren H.-Z., Shi X.-L. (2018). Lipometabolism and Glycometabolism in Liver Diseases. BioMed Res. Int..

[38] Sookoian S., Pirola C.J. (2015). Liver enzymes, metabolomics and genome-wide association studies: From systems biology to the personalized medicine. World J. Gastroenterol..

[39] Zheng J., Tian X., Xu B., Yuan F., Gong J., Yang Z. (2020). Collagen Peptides from Swim Bladders of Giant Croaker (*Nibea japonica*) and Their Protective Effects against H_2_O_2_-Induced Oxidative Damage toward Human Umbilical Vein Endothelial Cells. Mar. Drugs.

[40] Melekoglu R., Ciftci O., Eraslan S., Cetin A., Basak N. (2018). Beneficial effects of curcumin and capsaicin on cyclophosphamide-induced premature ovarian failure in a rat model. J. Ovarian Res..

[41] Tejero J., Shiva S., Gladwin M.T. (2019). Sources of Vascular Nitric Oxide and Reactive Oxygen Species and Their Regulation. Physiol. Rev..

[42] Dai C., Xiao X., Li D., Tun S., Wang Y., Velkov T., Tang S. (2018). Chloroquine ameliorates carbon tetrachloride-induced acute liver injury in mice via the concomitant inhibition of inflammation and induction of apoptosis. Cell Death Dis..

[43] Kany S., Vollrath J.T., Relja B. (2019). Cytokines in Inflammatory Disease. Int. J. Mol. Sci..

[44] Cavalcanti M.R.M., Passos F.R.S., Monteiro B.S., Gandhi S.R., Heimfarth L., Lima B.S., Nascimento Y.M., Duarte M.C., Araujo A.A.S., Menezes I.R.A. (2021). HPLC-DAD-UV analysis, anti-inflammatory and anti-neuropathic effects of methanolic extract of Sideritis bilgeriana (lamiaceae) by NF-κB, TNF-α, IL-1β and IL-6 involvement. J. Ethnopharmacol..

[45] Negash A.A., Ramos H.J., Crochet N., Lau D.T.Y., Doehle B., Papic N., Delker D.A., Jo J., Bertoletti A., Hagedorn C.H. (2013). IL-1β production through the NLRP3 inflammasome by hepatic macrophages links hepatitis C virus infection with liver inflammation and disease. PLoS Pathog..

[46] Gao R.Y., Wang M., Liu Q., Feng D., Wen Y., Xia Y., Colgan S.P., Eltzschig H.K., Ju C. (2020). Hypoxia-Inducible Factor-2α Reprograms Liver Macrophages to Protect Against Acute Liver Injury Through the Production of Interleukin-6. Hepatology.

[47] Liu A., Sun Y., Wang X., Ihsan A., Tao Y., Chen D., Peng D., Wu Q., Wang X., Yuan Z. (2019). DNA methylation is involved in pro-inflammatory cytokines expression in T-2 toxin-induced liver injury. Food Chem. Toxicol. Int. J. Publ. Br. Ind. Biol. Res. Assoc..

[48] Liu Y., Wen P.-H., Zhang X.-X., Dai Y., He Q. (2018). Breviscapine ameliorates CCl4-induced liver injury in mice through inhibiting inflammatory apoptotic response and ROS generation. Int. J. Mol. Med..

[49] He Y., Hwang S., Ahmed Y.A., Feng D., Li N., Ribeiro M., Lafdil F., Kisseleva T., Szabo G., Gao B. (2021). Immunopathobiology and therapeutic targets related to cytokines in liver diseases. Cell. Mol. Immunol..

[50] Carvalho A.M.S., Heimfarth L., Pereira E.W.M., Oliveira F.S., Menezes I.R.A., Coutinho H.D.M., Picot L., Antoniolli A.R., Quintans J.S.S., Quintans-Júnior L.J. (2020). Phytol, a Chlorophyll Component, Produces Antihyperalgesic, Anti-inflammatory, and Antiarthritic Effects: Possible NFκB Pathway Involvement and Reduced Levels of the Proinflammatory Cytokines TNF-α and IL-6. J. Nat. Prod..

[51] An S.Y., Petrescu A.D., DeMorrow S. (2021). Targeting Certain Interleukins as Novel Treatment Options for Liver Fibrosis. Front. Pharmacol..

[52] Sun X., Wu A., Law B.Y.K., Liu C., Zeng W., Qiu A.C.L., Han Y., He Y., Wong V.K.W. (2020). The active components derived from Penthorum chinense Pursh protect against oxidative-stress-induced vascular injury via autophagy induction. Free Radic. Biol. Med..

[53] Hammouda M.B., Ford A.E., Liu Y., Zhang J.Y. (2020). The JNK Signaling Pathway in Inflammatory Skin Disorders and Cancer. Cells.

[54] Coulthard L.R., White D.E., Jones D.L., McDermott M.F., Burchill S.A. (2009). p38MAPK: Stress responses from molecular mechanisms to therapeutics. Trends Mol. Med..

[55] He Y., She H., Zhang T., Xu H., Cheng L., Yepes M., Zhao Y., Mao Z. (2018). p38 MAPK inhibits autophagy and promotes microglial inflammatory responses by phosphorylating ULK1. J. Cell Biol..

[56] Nie Y., Wang Z., Chai G., Xiong Y., Li B., Zhang H., Xin R., Qian X., Tang Z., Wu J. (2019). Dehydrocostus Lactone Suppresses LPS-induced Acute Lung Injury and Macrophage Activation through NF-κB Signaling Pathway Mediated by p38 MAPK and Akt. Molecules.

[57] Sun S.C. (2017). The non-canonical NF-κB pathway in immunity and inflammation. Nat. Rev. Immunol..

[58] Liang W.-J., Yang H.-W., Liu H.-N., Qian W., Chen X.-L. (2020). HMGB1 upregulates NF-kB by inhibiting IKB-α and associates with diabetic retinopathy. Life Sci..

